# Effect of intracerebroventricular (ICV) injection of adrenomedullin and its interaction with NPY and CCK pathways on food intake regulation in neonatal layer-type chicks

**DOI:** 10.1016/j.psj.2024.103819

**Published:** 2024-05-06

**Authors:** Maryam Soleymani Zahed, Samad Alimohammadi, Shahin Hassanpour

**Affiliations:** ⁎ Section of Physiology, Department of Basic Sciences and Pathobiology, Faculty of Veterinary Medicine, Razi University, Kermanshah, Iran; † Section of Physiology, Department of Basic Sciences, Faculty of Veterinary Medicine, Science and Research Branch, Islamic Azad University, Tehran, Iran

**Keywords:** adrenomedullin, NPY, CCK, food intake, chicken

## Abstract

Adrenomedullin has various physiological roles including appetite regulation. The objective of present study was to determine the effects of ICV injection of adrenomedullin and its interaction with NPY and CCK receptors on food intake regulation. In experiment 1, chickens received ICV injection of saline and adrenomedullin (1, 2, and 3 nmol). In experiment 2, birds injected with saline, B5063 (NPY_1_ receptor antagonist, 1.25 µg), adrenomedullin (3 nmol) and co-injection of B5063+adrenomedullin. Experiments 3 to 5 were similar to experiment 2 and only SF22 (NPY_2_ receptor antagonist, 1.25 µg), SML0891 (NPY_5_ receptor antagonist, 1.25 µg) and CCK_4_ (1 nmol) were injected instead of B5063. In experiment 6, ICV injection of saline and CCK_8s_ (0.125, 0.25, and 0.5 nmol) were done. In experiment 7, chickens injected with saline, CCK_8s_ (0.125 nmol), adrenomedullin (3 nmol) and co-injection of CCK_8s_+adrenomedullin. After ICV injection, birds were returned to their individual cages immediately and cumulative food intake was measured at 30, 60, and 120 min after injection. Adrenomedullin (2 and 3 nmol) decreased food intake compared to control group (*P* < 0.05). Coinjection of B5063+adrenomedullin amplified hypophagic effect of adrenomedullin (*P* < 0.05). The ICV injection of the CCK_8s_ (0.25 and 0.5 nmol) reduced food intake (*P* < 0.05). Co-injection of the CCK_8s_+adrenomedullin significantly potentiated adrenomedullin-induced hypophagia (*P* < 0.05). Administration of the SF22, SML0891 and CCK_4_ had no effect on the anorexigenic response evoked by adrenomedullin (*P* > 0.05). These results suggested that the hypophagic effect of the adrenomedullin is mediated by NPY_1_ and CCK_8s_ receptors. However, our novel results should form the basis for future experiments.

## INTRODUCTION

Feeding behavior is a complex physiological process that can be influenced by external stimuli (such as environmental and dietary factors) as well as internal factors (such as digestive, hormonal, and neurological factors) (Zendehdel et al., 2020). The regulation of food intake and metabolism involves the involvement of neurotransmitters, hormones, and peptides, as well as the potential interaction of various neural pathways with hormones in the central nervous system (CNS) (Abot et al., 2018). Adrenomedullin is a peptide composed of 52 amino acids, which was initially discovered in the adrenal medulla and is widely distributed throughout the body, including various regions of the brain. It belongs to the calcitonin gene-related peptide (CGRP) family and exerts its effects on hypothalamic nuclei, such as the paraventricular nucleus (PVN) and supraoptic nucleus (SON) (Wang et al., 2014). The additional acknowledged individuals of the CGRP lineage include intermedin (IMD) and amylin (Ogoshi et al., 2006). Adrenomedullin has been detected in diverse animal species, such as domestic and farm mammals and birds (Zudaire et al., 2005; Martinez-Herrero and Martinez, 2016). Adrenomedullin exerts various physiological functions encompassing cardiovascular homeostasis (Kita and Kitamura, 2022), growth and development (Witlin et al., 2002), neurotransmission (Julian et al., 2005), a significant regulator in the immune response (Pedreno et al., 2014) and also in the digestive system (Martinez-Herrero and Martinez, 2016). Moreover, it has been established that adrenomedullin regulates food intake. For instance, the ICV injection of adrenomedullin impeded feeding in the rat by stimulating the CGRP receptors (Taylor et al., 1996). Similarly, after the ICV injection of adrenomedullin, the drinking behavior was diminished in rats (Murphy and Samson, 1995). Furthermore, it has been demonstrated that the administration of adrenomedullin centrally reduces the consumption of food but not water intake in broiler chicks (Wang et al., 2014). Additionally, anorexia induced by adrenomedullin has been documented in Japanese quail when administered centrally (Wang et al., 2021). Neuropeptide Y (NPY) is widely distributed throughout both the central and peripheral nervous systems (Gray and Morley, 1986). The arcuate nucleus of the hypothalamus (ARC) plays a crucial role in the regulation of food intake and body weight. Neurons expressing NPY are primarily detected in the ARC (Bi et al., 2012). It has been reported that the central injection of NPY significantly enhances food intake in avian species (Newmyer et al., 2013; McConn et al., 2018). Neuropeptide Y functions as an orexigenic factor and exerts its effects through NPY receptors, which belong to the family of G-protein-coupled receptors (GPCRs) (Yu et al., 2021). Among the subtypes of NPY receptors, NPY_1_, NPY_2_, and NPY_5_ are responsible for the regulation of feeding behavior and energy expenditure (Levens et al., 2004; Yousefvand et al., 2020). For example, there have been reports indicating that the administration of both NPY_1_ and NPY_5_ receptor antagonists (specifically, B5063 and SML0891, respectively) resulted in a reduction in food intake in broiler chickens in a dose-dependent manner, while the NPY_2_ receptor antagonist (SF22) caused an increase in food intake that was also dose-dependent (Yousefvand et al., 2019). Cholecystokinin (CCK), a neurohormone peptide present in the brains of both chickens and mammals, is one of the most prevalent ones (Vázquez-León et al., 2018). Various biologically active forms of CCK, such as CCK_4_, CCK_8_, CCK_8s_, CCK_33_, and CCK_58_, have been identified in different brain regions. Cholecystokinin interacts with specific receptors that have been categorized into 2 subtypes: CCK_A_ (CCK_1_) and CCK_B_ (CCK_2_) (Miyasaka and Funakoshi, 2003). In the brain, CCK is recognized as a neurotransmitter that suppresses feeding behavior. For instance, the administration of CCK in rats led to a suppression of their feeding behavior (Maniscalco et al., 2020). An anorectic response induced by CCK was observed in rats upon intraperitoneal injection of CCK (Akieda-Asai et al., 2014). In neonatal chickens, the ICV injection of CCK_8S_ exhibited a hypophagic effect similar to that of intraperitoneal administration. Conversely, CCK_4_ did not have any impact on food consumption (Tachibana et al., 2012). It appears that there is a functional interaction between adrenomedullin, NPY, and CCK. The central administration of adrenomedullin in Japanese quail increased the mRNA expression of proopiomelanocortin (POMC) and cocaine- and amphetamine-regulated transcript (CART) in the arcuate nucleus (ARC) of the hypothalamus (Wang et al., 2021). Hypothalamic interactions between NPY, POMC and CART neurons have also been demonstrated (Dhillo et al., 2002). A negative relation between NPY and CCK peptides has been found in the regulation of feeding behavior in rats (Gourch et al., 1990). In addition, a synergistic interaction between anorexigenic effects of amylin and CCK was proved in goldfish (Thavanathan and Volkoff, 2006). NPY inhibits CCK-stimulated exocrine pancreatic secretion in a dose-dependent manner (Pandiri, 2014). Also, CCK-induced increase in amylase release from pancreatic acini can be reversed by adrenomedullin administration (Tsuchida et al., 1999).

Based on findings in the previous literature and considering that adrenomedullin, NPY and CCK have interaction on a number of physiological processes, the present study was designed to determine the interaction of centrally administered adrenomedullin with NPY and CCK pathways on food intake regulation in neonatal layer-type chicks.

## MATERIALS AND METHODS

### Animals

In this particular study, a total of 308 one-day-old layer-type chickens were acquired from a local hatchery known as Morghak Company, located in Tehran, Iran. The aforementioned chickens were initially kept in groups for a duration of 2 d, after which they were randomly transferred to individual cages. During this time, the temperature in the cages was maintained at 30 ± 1 ºC, with a humidity level of 50 ± 2% (Olanrewaju et al., 2017). The chickens were provided with a commercial starter diet, which consisted of 21% crude protein and 2850 kcal/kg of metabolizable energy. This diet was sourced from Chineh Company, Tehran, Iran (Table 1). It is worth noting that all the birds in the study were given unrestricted access to food and fresh water throughout the entire duration. Three hours prior to the ICV injections, the chickens were deprived of food (FD3), while still being allowed to freely consume water. The experiments commenced when the chickens reached the age of 5 d. It should be noted that all the experimental procedures conducted in this study were approved by the Animal Ethics Committee of Razi University, with the approval number IR.RAZI.REC.1401.027. Furthermore, these procedures were carried out in accordance with the Guidelines for the Care and Use of Laboratory Animals in Research (Zimmermann, 1983).

**Table 1:** Ingredient and nutrient analysis of experimental diet.

Ingredient (%)		Nutrient analysis	
Corn	52.85	ME (kcal/g)	2850
Soybean meal, 48% CP	31.57	Crude protein (%)	21
Wheat	5	Linoleic acid (%)	1.69
Gluten meal, 61% CP	2.50	Crude fiber (%)	3.55
Wheat bran	2.47	Calcium (%)	1
Di-calcium phosphate	1.92	Available phosphorus (%)	0.5
Oyster shell	1.23	Sodium (%)	0.15
Soybean oil	1.00	Potassium (%)	0.96
Mineral premix	0.25	Chlorine (%)	0.17
Vitamin premix	0.25	Choline (%)	1.30
Sodium bicarbonate	0.21	Arginine (%)	1.14
Sodium chloride	0.20	Isoleucine (%)	0.73
Acidifier	0.15	Lysine (%)	1.21
DL-Methionine	0.10	Methionine (%)	0.49
Toxin binder	0.10	Methionine+Cystine (%)	0.83
L-Lysine HCl	0.05	Threonine (%)	0.70
Vitamin D3	0.1	Tryptophan (%)	0.20
Multi enzyme	0.05	Valine (%)	0.78

### Experimental Drugs

Adrenomedullin, B5063 (NPY_1_ receptor antagonist), SF22 (NPY_2_ receptor antagonist), SML0891 (NPY_5_ receptor antagonist), CCK (30-33) (also known as CCK_4_), CCK (26-33) (commonly referred to as Sulfated CCK_8_ or CCK_8s_), and Evans Blue were all obtained from Sigma Co. (Sigma, Saint Louis, MO). To prepare the medications for administration, they were dissolved in a 0.1% Evans Blue solution, which itself was prepared in a 0.85% saline solution at a ratio of 1/250. For the control group, saline containing Evans Blue was utilized (Alimohammadi et al., 2015).

### ICV Injection Procedures

Prior to each treatment, the chicks were weighed, and based on their body weight (BW), they were assigned to experimental groups. This allocation was done in such a way that the average weight among the treatment groups was as uniform as possible. The ICV injections were carried out using a microsyringe (Hamilton, Switzerland). It is important to note that the injections were performed without the use of anesthesia, following the techniques described in previous studies (Davis et al., 1979; Furuse et al., 1997). To facilitate this technique, the chick's head was held using an acrylic device, with the bill holder positioned at a 45º angle, and the calvarium parallel to the table surface. An orifice was created in a plate, which was then placed directly above the skull, specifically above the right lateral ventricle (Van Tienhoven and Juhasz, 1962). The microsyringe was inserted into the right ventricle through this orifice, with the tip of the needle penetrating only 4 mm beneath the skin of the skull. Each chick received an ICV injection, either of the vehicle solution or the drug solution, in a volume of 10 μL. It is worth mentioning that previous studies have demonstrated that this injection method does not induce any physiological stress in neonatal chicks (Saito et al., 2005). At the end of the experiments, the chicks were anesthetized. The state of anesthesia was ascertained through the nonexistence of reflexes, such as lack of reaction to external stimuli. Then, the chicks were sacrificed by decapitation in order to verify the accuracy of the injection placement. This was done by confirming the presence of Evans Blue dye in the frozen brain tissue, which was achieved by slicing the tissue. It is important to mention that only data from individual chicks were used for analysis if the lateral ventricle showed the presence of Evans Blue color. Lastly, all experimental procedures were conducted between 8:00 am and 3:30 pm (Alimohammadi et al., 2015).

### Feeding Experiments

In this study, a total of 7 experiments were conducted on each of the 4 treatment groups. Each group consisted of a minimum of 11 neonatal chicks, resulting in a total of 44 chicks in each experiment. The procedures for the treatments in the experiments can be found in Table 2. The purpose of experiment 1 was to examine the impact of ICV injection of adrenomedullin at various doses (1, 2, and 3 nmol) on the food intake of FD3 chickens. In experiment 2, the chicks were subjected to ICV injections of saline, B5063 (1.25 µg), adrenomedullin (3 nmol), and a combination of B5063 and adrenomedullin. Similarly, in experiment 3, the chickens received ICV injections of saline, SF22 (1.25 µg), adrenomedullin (3 nmol), and a combination of SF22 and adrenomedullin. Experiment 4 involved ICV injections of saline, SML0891 (1.25 µg), adrenomedullin (3 nmol), and the co-administration of SML0891 and adrenomedullin in the chicks. In experiment 5, the chicks were ICV injected with saline, CCK_4_ (1 nmol), adrenomedullin (3 nmol), and a combination of CCK_4_ and adrenomedullin. Experiment 6 entailed ICV injections of CCK_8s_ at different doses (0.125 nmol, 0.25 nmol, and 0.5 nmol) in the chicks. Lastly, in experiment 7, the chicks were ICV injected with saline, CCK_8s_ (0.125 nmol), adrenomedullin (3 nmol), and a combination of CCK_8s_ and adrenomedullin. Following the ICV injections, the chicks were immediately returned to their individual cages. Fresh water and food were provided, and the cumulative food intake (in grams) was measured at 30, 60, and 120 min after the injection. To account for the potential influence of body weight on food consumption, the food intake was expressed as a percentage of the body weight. Each bird was only used once in each experimental group. The doses of the drugs used for ICV injection were determined based on previous reports (Wang et al., 2014; Rajaei et al., 2022; Jelokhani et al., 2022) and pilot studies that have not been published.

**Table 2:** Treatments procedure in experiments 1–7.

Experiment 1	ICV injection
Treatment groups	
A	Control Solution
B	Adrenomedullin (1 nmol)
C	Adrenomedullin (2 nmol)
D	Adrenomedullin (3 nmol)

Experiment 2	ICV injection

Treatment groups	
A	Control Solution
B	B5063 (NPY_1_ receptor antagonist, 1.25 µg)
C	Adrenomedullin (3 nmol)
D	B5063+Adrenomedullin

Experiment 3	ICV injection

Treatment groups	
A	Control Solution
B	SF22 (NPY_2_ receptor antagonist, 1.25 µg)
C	Adrenomedullin (3 nmol)
D	SF22+Adrenomedullin

Experiment 4	ICV injection

Treatment groups	
A	Control Solution
B	SML0891 (NPY_5_ receptor antagonist, 1.25 µg)
C	Adrenomedullin (3 nmol)
D	SML0891+Adrenomedullin

Experiment 5	ICV injection

Treatment groups	
A	Control Solution
B	CCK_4_ (1 nmol)
C	Adrenomedullin (3 nmol)
D	CCK_4_+Adrenomedullin

Experiment 6	ICV injection

Treatment groups	
A	Control Solution
B	CCK_8s_ (0.125 nmol)
C	CCK_8s_ (0.25 nmol)
D	CCK_8s_ (0.5 nmol)

Experiment 7	ICV injection

Treatment groups	
A	Control Solution
B	CCK_8s_ (0.125 nmol)
C	Adrenomedullin (3 nmol)
D	CCK_8s_+Adrenomedullin

### Statistical Analysis

Data were presented in the form of mean ± SEM. The analysis of cumulative food intake, expressed as a percentage of body weight, was conducted using a 2-way analysis of variance (ANOVA) with repeated measures. This analysis was performed using the SPSS software version 21 for Windows (SPSS, Inc., Chicago, IL). For treatments that showed a significant main effect in the ANOVA, means were compared using the Tukey-Kramer test. A significance level of *P* < 0.05 was used to indicate the presence of significant differences between the treatments.

## RESULTS

**Figure 1:**
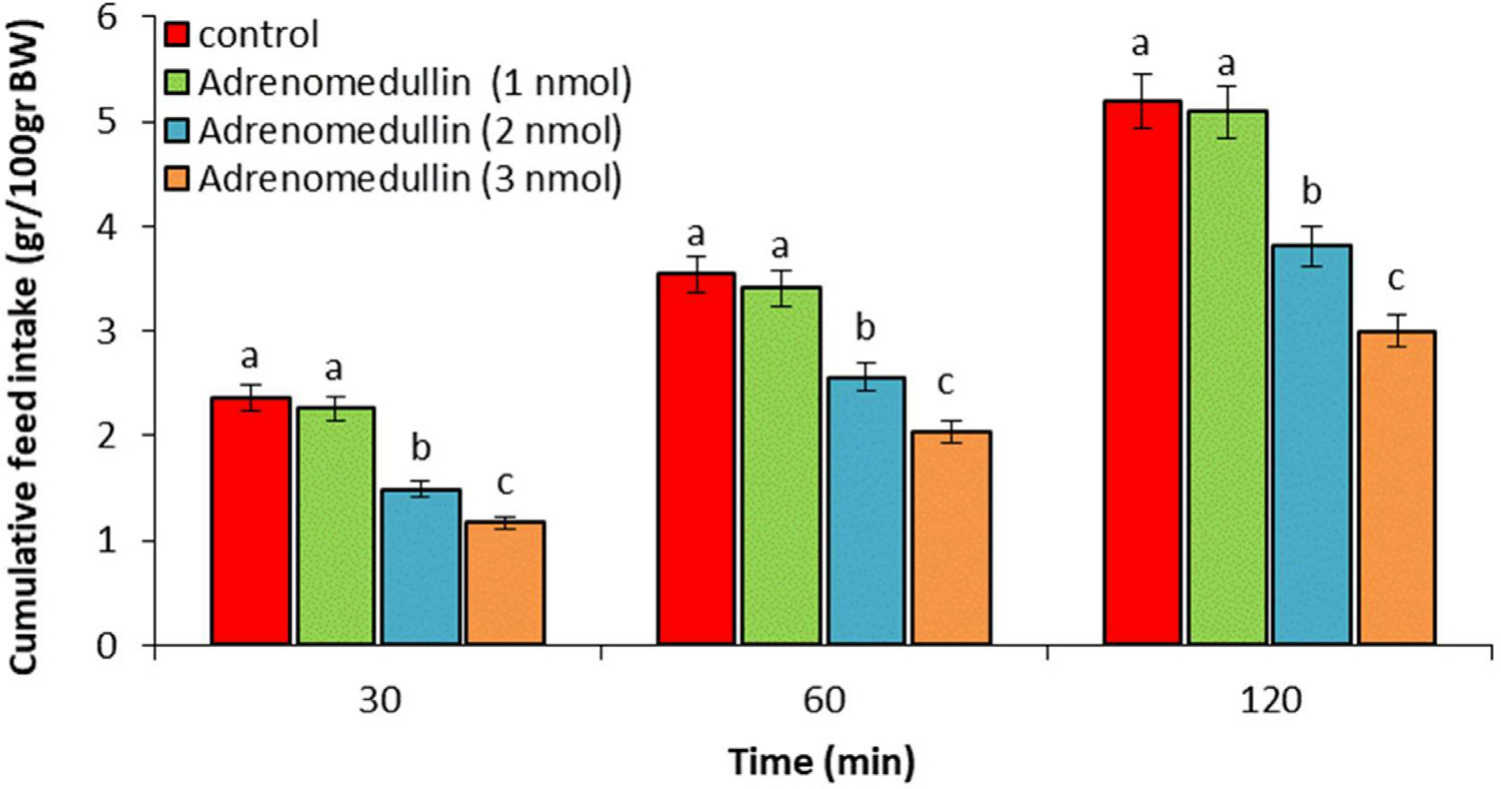
Effect of intracerebroventricular injection of control solution and different levels of the adrenomedullin (1, 2, and 3 nmol) on cumulative food intake (gr/100gr BW) in neonatal layer-type chicks. Data are expressed as mean ± SEM. Different letters (a, b and c) indicate significant differences between treatments at each time (*P* < 0.05).

**Figure 2:**
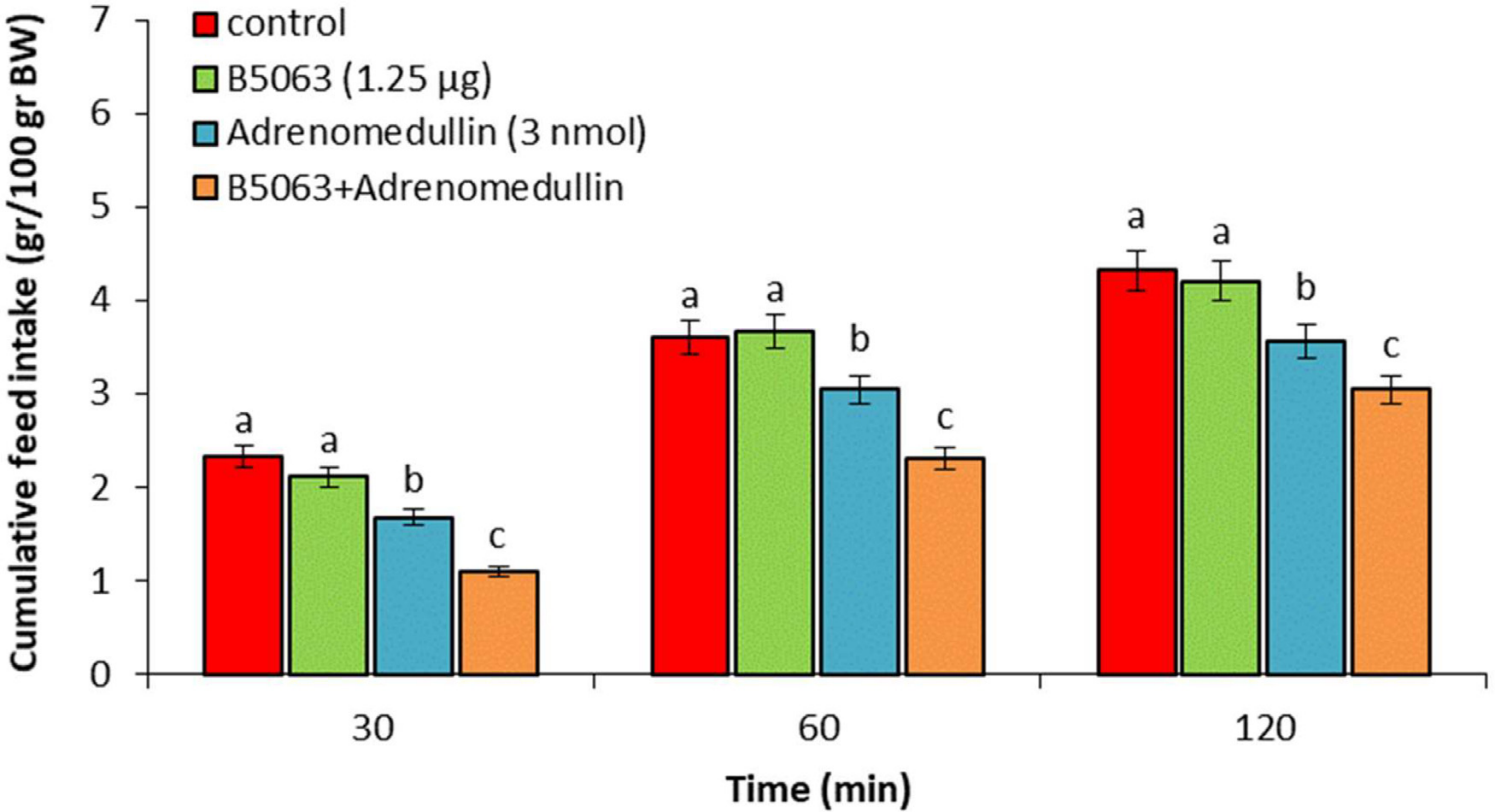
Effect of intracerebroventricular injection of control solution, B5063 (NPY_1_ receptor antagonist, 1.25 µg), adrenomedullin (3 nmol) and a combination of B5063 plus adrenomedullin on cumulative food intake (gr/100gr BW) in neonatal layer-type chicks. Data are expressed as mean ± SEM. Different letters (a, b and c) indicate significant differences between treatments at each time (*P* < 0.05).

**Figure 3:**
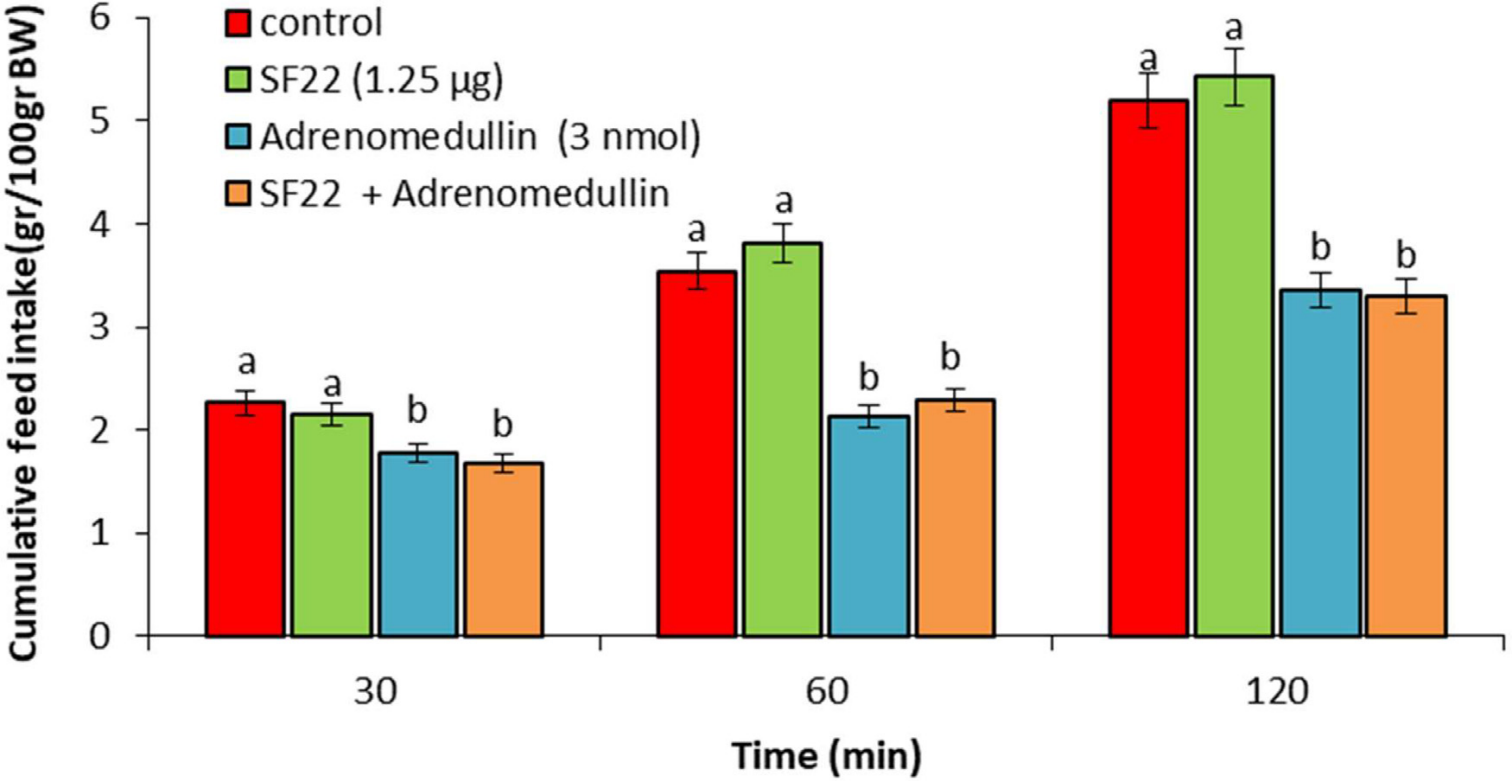
Effect of intracerebroventricular injection of control solution, SF22 (NPY_2_ receptor antagonist, 1.25 µg), adrenomedullin (3 nmol) and a combination of SF22 plus adrenomedullin on cumulative food intake (gr/100gr BW) in neonatal layer-type chicks. Data are expressed as mean ± SEM. Different letters (a and b) indicate significant differences between treatments at each time (*P* < 0.05).

**Figure 4:**
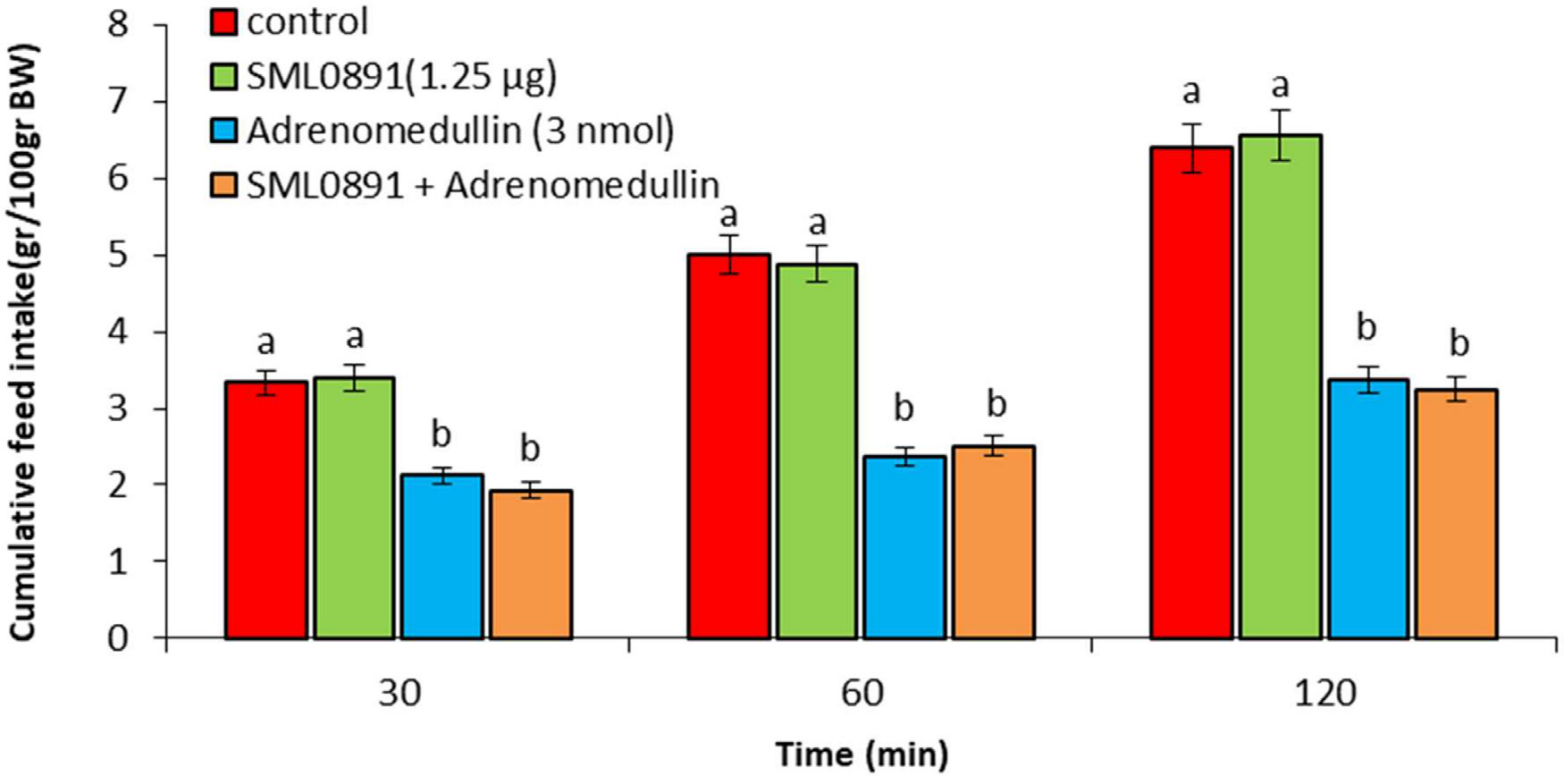
Effect of intracerebroventricular injection of control solution, SML0891 (NPY_5_ receptor antagonist, 1.25 µg), adrenomedullin (3 nmol) and a combination of SML0891 plus adrenomedullin on cumulative food intake (gr/100gr BW) in neonatal layer-type chicks. Data are expressed as mean ± SEM. Different letters (a and b) indicate significant differences between treatments at each time (*P* < 0.05).

**Figure 5:**
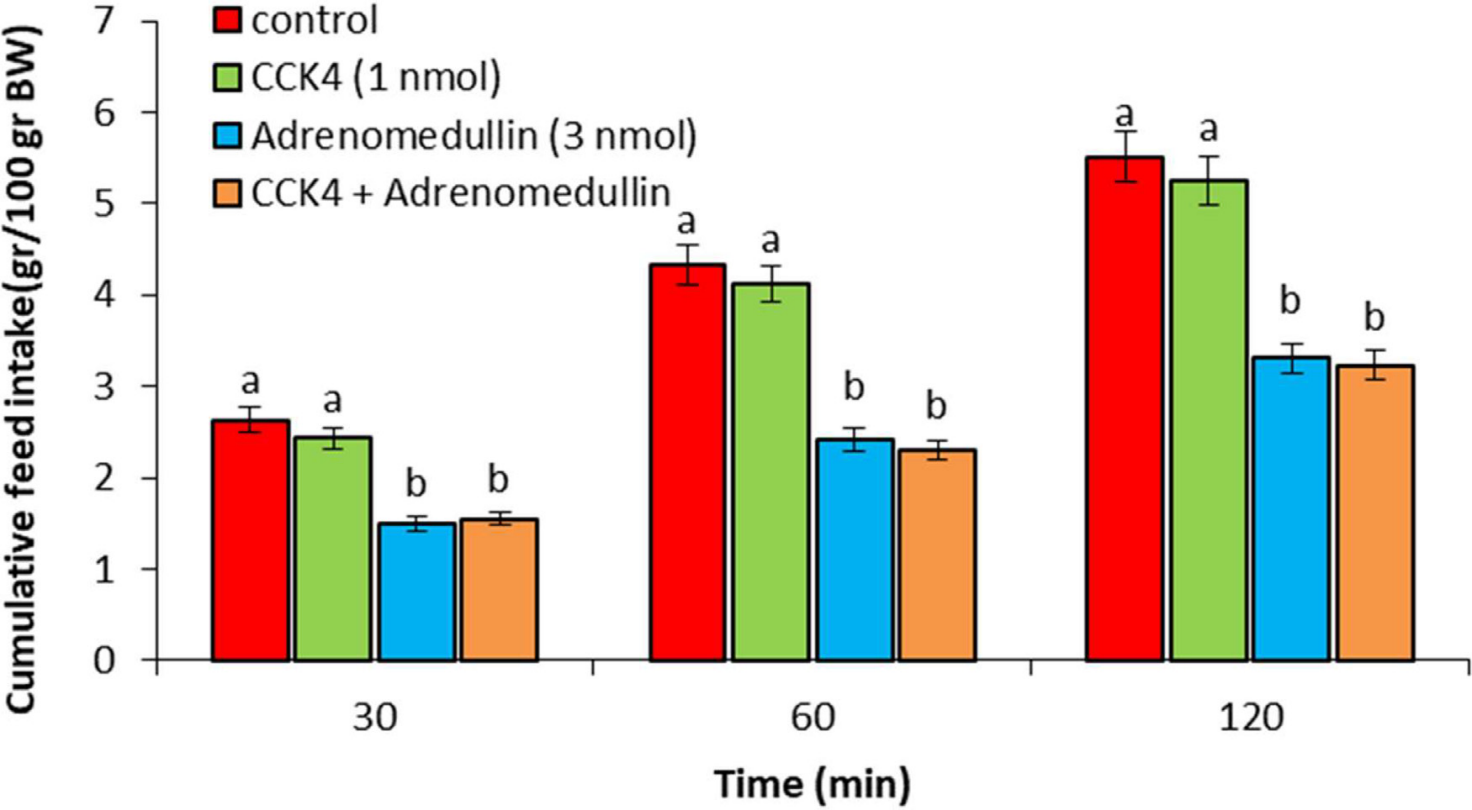
Effect of intracerebroventricular injection of control solution, CCK_4_ (1 nmol), adrenomedullin (3 nmol) and a combination of CCK_4_ plus adrenomedullin on cumulative food intake (gr/100gr BW) in neonatal layer-type chicks. Data are expressed as mean ± SEM. Different letters (a and b) indicate significant differences between treatments at each time (*P* < 0.05).

**Figure 6:**
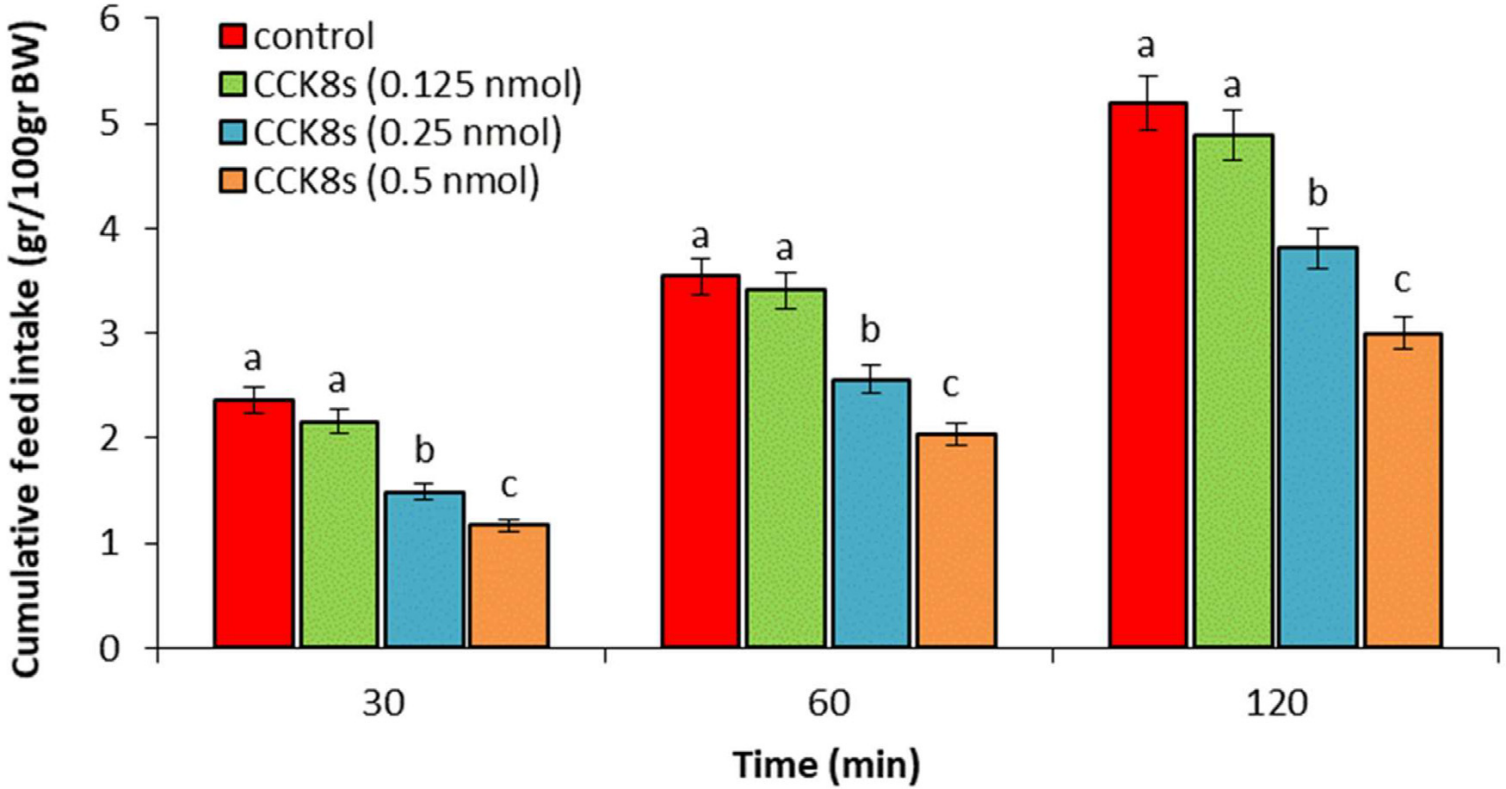
Effect of intracerebroventricular injection of control solution and different levels of the CCK_8s_ (0.125, 0.25, and 0.5 nmol) on cumulative food intake (gr/100gr BW) in neonatal layer-type chicks. Data are expressed as mean ± SEM. Different letters (a, b, and c) indicate significant differences between treatments at each time (*P* < 0.05).

**Figure 7:**
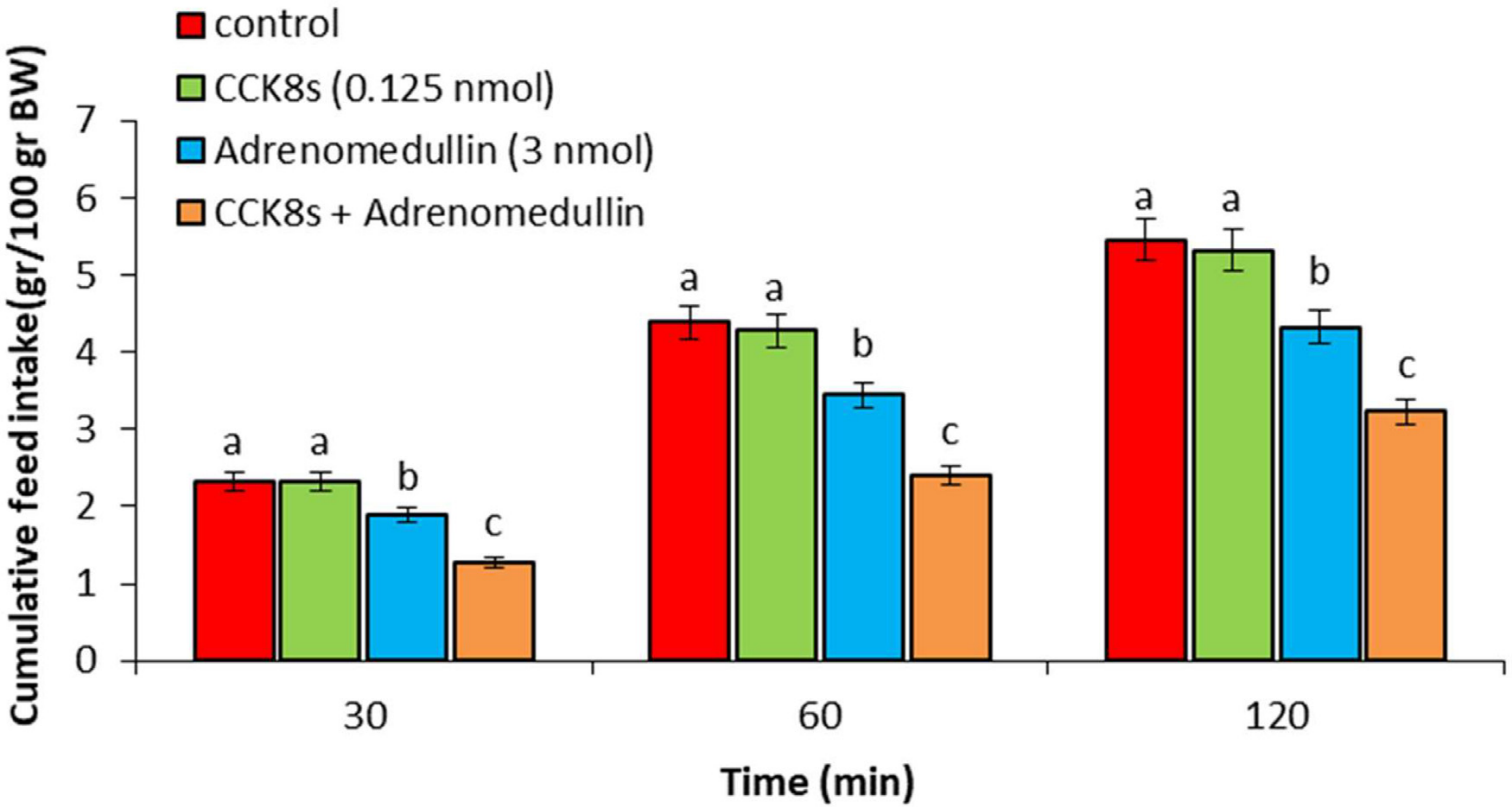
Effect of intracerebroventricular injection of control solution, CCK_8s_ (0.125 nmol), adrenomedullin (3 nmol) and a combination of CCK_8s_ plus adrenomedullin on cumulative food intake (gr/100gr BW) in neonatal layer-type chicks. Data are expressed as mean ± SEM. Different letters (a, b, and c) indicate significant differences between treatments at each time (*P* < 0.05).

In experiment 1, ICV injection of adrenomedullin at doses of 2 and 3 nmol resulted in a significant dose-dependent decrease in food intake over the entire 120-min observation period (*P* < 0.05). However, no significant difference was observed between the 1 nmol dose of adrenomedullin and the control group (*P* > 0.05) (Figure 1). In experiment 2, ICV injection of B5063 at a dose of 1.25 µg had no significant effect on food intake compared to the control group (*P* > 0.05). Adrenomedullin at a dose of 3 nmol significantly decreased food intake at 30, 60, and 120 min postinjection compared to the control group (*P* < 0.05). The hypophagic effect of adrenomedullin was significantly enhanced by co-injection of B5063 and adrenomedullin in FD3 chickens (*P* < 0.05) (Figure 2). In experiment 3, ICV injection of SF22 at a dose of 1.25 µg had no significant effect on food consumption compared to the control group (*P* > 0.05). Adrenomedullin at a dose of 3 nmol was associated with decreased food intake at 30, 60, and 120 min postinjection compared to the control group (*P* < 0.05). The hypophagic effect of adrenomedullin was not affected by co-injection of SF22 and adrenomedullin in FD3 chickens (*P* > 0.05) (Figure 3). In experiment 4, ICV injection of SML0891 at a dose of 1.25 µg had no significant effect on food intake compared to the control group (*P* > 0.05). Adrenomedullin at a dose of 3 nmol was associated with decreased food intake at 30, 60, and 120 min postinjection compared to the control group (*P* < 0.05). Coinjection of SML0891 and adrenomedullin did not alter the adrenomedullin-induced hypophagia in FD3 chickens (P>0.05) (Figure 4). In experiment 5, no significant change in food consumption was observed after ICV injection of CCK_4_ at a dose of 1 nmol compared to the control group (*P* > 0.05). Adrenomedullin at a dose of 3 nmol decreased food intake up to 120 min after ICV administration compared to the control group (*P* < 0.05). Coinjection of CCK_4_ and adrenomedullin did not reverse the adrenomedullin-induced reduction in food intake in FD3 chickens (*P* > 0.05) (Figure 5). In experiment 6, CCK_8s_ at a dose of 0.125 nmol had no effect on food intake compared to the control group (*P* > 0.05); however, doses of 0.25 and 0.5 nmol of CCK_8s_ significantly decreased cumulative food intake in a dose-dependent manner compared to the control group (*P* < 0.05) (Figure 6). In experiment 7, ICV injection of CCK_8s_ at a dose of 0.125 nmol had no significant effect on food intake compared to the control group (*P* > 0.05). Adrenomedullin at a dose of 3 nmol decreased food intake up to 120 min after ICV administration compared to the control group (*P* < 0.05). The hypophagia induced by adrenomedullin was potentiated by co-injection of CCK_8s_ and adrenomedullin in FD3 chickens (*P* < 0.05) (Figure 7).

## DISCUSSION

To the best of our knowledge, this is the initial report that illustrates the impact of ICV injection of adrenomedullin and its potential interaction with NPY and CCK receptors on the regulation of food intake in layer-type chicks. Based on the outcomes of experiment 1, ICV injection of adrenomedullin led to a reduction in cumulative food intake in a dose-dependent manner in FD3 chickens, which aligns with previous research conducted on rats (Taylor et al., 1996), mice (Bech et al., 2019), broiler chicks (Wang et al., 2014), and Japanese quail (Wang et al., 2021). Interestingly, the influence of ICV injection of adrenomedullin on feeding behavior in goldfish was found to be negligible. This could potentially be attributed to the decreased binding affinity of human adrenomedullin to the goldfish receptor (Martinez-Alvarez et al., 2009). However, it is worth noting that the human peptide displayed a potent anorexigenic effect in the current study, as well as in other species mentioned earlier. Another member of the CGRP family, amylin, was found to induce anorexigenic effects through the hypothalamus and brain stem in chicks (Cline et al., 2008). The precise mechanisms by which adrenomedullin reduces food intake have not been fully elucidated. Nonetheless, previous studies have shed light on the inhibitory action of adrenomedullin. The anorectic property of adrenomedullin is attributed, at least in part, to the direct activation of CGRP receptors in the chick brain, leading to an increased sense of satiety (Wang et al., 2014). It has been reported that ICV injection of adrenomedullin resulted in elevated hypothalamic c-Fos immunoreactivity within the arcuate nucleus, which serves as an indicator of functional marker of activated neurons. Furthermore, mRNA expression of POMC and CART in the arcuate nucleus was found to be increased in adrenomedullin-treated quail (Wang et al., 2021).

In the present investigation, the concurrent injection of B5063 (an antagonist of the NPY_1_ receptor) and adrenomedullin augmented the hypophagic effect of adrenomedullin. Nevertheless, there was no correlation observed between adrenomedullin and the NPY_2_ and NPY_5_ receptors in neonatal layer-type chicks. Neuropeptide Y and its various subtypes of receptors have been demonstrated to play a role in the regulation of feeding behavior (Newmyer et al., 2013). In the context of chickens, it has been proven that NPY stimulates food consumption. Conversely, the administration of B5063 and SML0891, at doses of 2.5 and 5 μg, respectively, resulted in a dose-dependent decrease in food intake in newly hatched chickens (5-day-old chickens). On the other hand, SF22, when administered at doses of 2.5 and 5 μg, led to an increase in feed intake (Yousefvand et al., 2019).

The regulation of food intake is governed by intricate interactions among numerous neurotransmitters within the CNS. The intracerebroventricular injection of adrenomedullin in neonatal layer-type chicks led to a reduction in food intake for 120 minutes following the injection, accompanied by an elevated mRNA expression of anorectic neurotransmitters CART and POMC in the arcuate nucleus of the hypothalamus (Wang et al., 2021). In chickens, neurotransmitters that decrease food intake may rely on the corticotropin releasing factor (CRF) system. Indications have emerged suggesting that CRF neurons receive input from calcitonin gene-related peptide (CGRP) nerve terminals, thereby supporting the notion that CRF and CGRP interact to regulate feeding response (Martinez et al., 1997). However, due to the limitations of the present study, we were unable to determine the interaction between adrenomedullin and the CRF system in the regulation of food intake in chicks. While a relationship between the CGRP family and other factors has been established, the interaction between adrenomedullin and NPY in the inhibition of food intake by adrenomedullin has not yet been reported.

Another discovery from our investigation revealed that the co-administration of CCK_4_ and adrenomedullin did not reverse the reduction in food intake induced by adrenomedullin in chickens. Moreover, ICV administration of CCK_8s_ at higher doses resulted in a decrease in food consumption in chickens, suggesting that CCK may function as one of the anorexigenic mediators in the brain of layer chicks. This finding aligns with previous studies that demonstrated a decrease in food intake in broiler chicks (Covasa and Forbes, 1994) and layer chicks (Tachibana et al., 2012) when exposed to CCK_8s_. Exogenous CCK has been shown to induce satiation and trigger the complete pattern of satiety behavior in rodents, primates, and humans. It has been proposed that the anorexigenic effects of CCK are influenced by metabolic status and the presence or absence of other factors that regulate food intake (Cawthon and de La Serre, 2021). CCK is involved in regulating the sensitivity of vagal afferent neurons (VAN) to signals of satiation and hunger. Additionally, the presence of a gastric load enhances the reduction in food intake induced by CCK (Wu et al., 2020). Activation of VAN by CCK leads to the expression of other anorexigenic signals, including leptin, in the nucleus tractus solitarius (NTS) (Peters et al., 2006). Furthermore, our study revealed that the co-administration of CCK_8s_ and adrenomedullin augmented the hypophagic effect of adrenomedullin, demonstrating a synergistic interaction between adrenomedullin and CCK_8s_ in the regulation of feeding behavior. This finding is consistent with previous reports that described a strong interaction between amylin (an analog of adrenomedullin) and CCK in reducing food intake in goldfish (Thavanathan and Volkoff, 2006) and mice (Mollet et al., 2003). As there is no existing evidence regarding chicks, we are unable to compare our findings with those from previous studies.

## CONCLUSIONS

In summary, our findings suggest that the hypophagia induced by adrenomedullin in neonatal layer-type chicks may be regulated by the pathways of NPY_1_ and CCK_8s_, rather than the pathways of NPY_2_, NPY_5_, and CCK_4_. Nonetheless, these innovative results should serve as the foundation for future experiments, and further investigations are necessary to clarify the underlying cellular and molecular signaling pathways that connect adrenomedullin with NPY and CCK pathways in the regulation of feeding behavior in poultry.
